# Evaluation of nurses’ attitudes and behaviors regarding narcotic drug safety and addiction: a descriptive cross-sectional study

**DOI:** 10.1186/s12912-024-02109-2

**Published:** 2024-06-26

**Authors:** Ayten Kaya, Zila Özlem Kirbaş, Suhule Tepe Medin

**Affiliations:** 1 İkizce Vocational School, Ordu University, Ordu, Türkiye; 2 Faculty of Health Sciences, Department of Nursing, Bayburt University, Bayburt, Türkiye; 3 Ordu Public Hospital, Ordu, Türkiye

**Keywords:** Narcotic drug safety, Substance abuse, Attitude, Nurse

## Abstract

**Background:**

By evaluating nurses’ attitudes and behaviors regarding narcotic drug safety and addiction, effective strategies need to be developed for combating addiction in healthcare institutions. This study, aimed at providing an insight into patient and staff safety issues through the formulation of health policies, aimed to evaluate nurses’ attitudes and behaviors regarding narcotic drug safety and addiction.

**Methods:**

The study was conducted in a descriptive cross-sectional design. It was carried out with 191 nurses in a public hospital between March 2023 and August 2023. Data were collected through face-to-face interviews, gathering socio-demographic information and utilizing the Addictive Substance Attitude Scale. The data were analyzed using independent sample t-tests, one-way ANOVA tests, and regression analysis (*P* < .001 and *p* < .05).

**Results:**

The average age of the participants was determined to be 36.58 ± 8.40. It was reported by 85.3% of nurses that narcotic drug follow-ups in their units were conducted according to procedures. In the study, it was found that 63.9% of nurses did not know the procedure to be followed towards a healthcare professional identified as a narcotic substance addict. The total mean score of the Addictive Substance Attitude Scale of nurses participating in the study was 74.27 ± 14.70. A significant difference was found between the total scores of the scale and the level of education of nurses, the follow-up status of the drugs in the unit where they work, their status of receiving narcotic drug addiction training, and their routine use of the half-dose drug disposal form (*p* < .05).

**Conclusions:**

The findings of this study underscore the importance of evaluating nurses’ attitudes and behaviors regarding narcotic drug safety and addiction. These results indicate the need for nursing administrators, particularly in terms of patient and staff safety, to adopt more effective policies and strategies.

## Background

Addiction is the continued use of a substance despite the fact that it causes mental, physical or social problems, the inability to quit despite the desire to quit, and the inability to stop the desire to take the substance [1]. There are many factors that affect the addiction process. The person’s genetic structure, gender, existing mental illnesses, personality traits such as impulsivity and novelty-seeking, the environment in which one lives, chaotic home environment, substance use by parents in the family, lack of appropriate parental supervision, negative experiences in childhood, influence of friends, starting to use substances at an early age, and the properties of the substance itself affect the development of addiction. In addition to these, the workload of healthcare professionals, sleep patterns disorders, long working hours, and mobbing to which they are exposed on the job are also factors that affect the addiction process [2].

Healthcare institutions are places where opioid-type painkillers or anesthetic substances are concentrated. The presence of drugs that cause addiction in health institutions and the easy access of nurses to drugs pose a risk of substance use among nurses. In addition to intense work stress, changing working conditions, and addiction to these drugs can develop after any trauma or surgery. Although it is known that there are employees who use addictive drugs in health institutions, official statistics for this situation are not yet available. Healthcare professionals can hide this addiction for reasons such as fear of losing their job, fear of loss of prestige, or self-treatment [3]. Similarly, corporate managers can ignore such situations in order to prevent the loss of prestige of their institutions. Nurses and other healthcare professionals may prefer to adopt an attitude as if it does not happen at all, especially in cases of anesthetic and opioid-type drug addiction. There are no official data on how common anesthetic substance addiction is among healthcare professionals in Türkiye. In addition to studies showing that the incidence of substance addiction in healthcare professionals is the same as in society, there is also data showing that healthcare professionals are more prone to addiction to anesthetic and narcotic drugs, especially in clinical settings [4, 5]. Studies conducted around the world show that healthcare professionals are more prone to substance addiction. In the USA, 62% of residency program directors reported that at least one trainee had a substance abuse problem and an alarming increase in the incidence was noted [6, 7].

Healthcare institutions are places where opioid-type pain relievers or anesthetic substances are concentrated. The presence of drugs that can lead to addiction in healthcare institutions and nurses’ easy access to these drugs pose a risk for substance use among nurses. Intense work stress, along with changing working conditions following any trauma or surgery, can lead to addiction to these drugs. While it is known that there are employees in healthcare institutions who use addictive substances, official statistics regarding this issue are not yet available. Healthcare workers may conceal this addiction due to fear of losing their job, fear of losing prestige, or self-treatment. Similarly, institutional managers may turn a blind eye to such situations in order to prevent prestige loss for their institutions. Nurses and other healthcare workers may prefer to adopt an attitude as if nothing is happening, especially in cases of anesthesia and opioid-type substance addiction. In Türkiye, there is no official data available on the prevalence of anesthetic substance addiction among healthcare workers. In addition to studies indicating that the frequency of substance addiction among healthcare workers is similar to that in the general population, there is also data suggesting that healthcare workers, especially in clinical settings, are more prone to anesthesia and narcotic drug addiction. Research conducted worldwide indicates that healthcare workers are more susceptible to substance addiction. In the United States, 62% of residency program directors reported at least one trainee experiencing substance addiction issues, with a concerning increase in cases noted [6, 7].

Nursing is a professional occupation aimed at protecting and promoting the health of individuals, families, and communities, as well as restoring their physical, mental, and social integrity in case of disruption, and ensuring their return to their former state [8]. Nurses play important roles in combating addiction by taking preventive measures, providing support to patients, and managing treatment processes. Within these roles, they conduct activities such as patient education, management of support groups, assessment of addiction-related risk factors, and planning appropriate interventions. Nurses working collaboratively with the healthcare team in the prevention and treatment process of substance addiction may encounter excessive presence of narcotic drugs in their work environments and may come across addicted colleagues due to heavy work conditions or social reasons. While data on substance use among nurses are insufficient, research suggests that substance use among nurses is similar to the general population [9, 10].

Professional occupations are responsible for ensuring that their professions are delivered in accordance with ethical standards. Particularly, nurses are obligated to adhere to ethical principles while providing care, to protect public health, and to maintain the image of nursing. Recognizing their colleagues’ substance use, and protecting patients and the professional image are professional responsibilities. Hospital administrators’ failure to address or intervene to protect addicted employees can lead to worse outcomes.

Nurses have easier access to opioid analgesics and anesthetic substances compared to other members of society. This access can be facilitated by diverting medication intended for patients for personal use, taking leftover doses, or directly obtaining drugs from areas where narcotic substances are stored. In this regard, narcotic drug addiction not only impacts the health of the addicted nurses themselves but also compromises the health of the patients under their care [11]. To combat drug abuse, the Ministry of Health of the Republic of Türkiye has mandated the standardization of narcotic drug management within the framework of Health Quality Standards (HCS) [12]. Accordingly, all hospitals have been directed to regulate the administration of narcotic drugs and have implemented these regulations in their units. These comprehensive guidelines outline the procedures for ordering narcotics, obtaining them from the pharmacy, administering them to patients, recording the process, and storing the drugs in locked cabinets. Despite all these precautions, narcotic addiction and deaths resulting from it can still be encountered in hospitals. The easy access to narcotic drugs poses a risk of substance abuse among nurses. This issue, often overlooked and ignored in healthcare institutions, is of paramount importance for nursing due to its adverse effects on patient safety, public trust in healthcare services, and the nursing profession’s image. The attitudes of nurses towards addicted colleagues are of paramount importance in early detection, reporting, and intervention to protect patients from harm and to assist colleagues in their recovery. The attitude of colleagues is crucial in identifying nurses suspected of addiction, guiding them towards medical assistance, and supporting them during treatment and rehabilitation stages. Research examining nurses’ attitudes towards individuals who use substances has revealed that nurses exhibit similar negative attitudes and reactions towards addicted individuals as seen in society [13, 14].

Ford et al. (2008) demonstrated that as nurses’ biases against individuals using substances increased, their therapeutic behaviors decreased [15]. Stigmatization and exclusion of individuals identified as addicted by their colleagues erode trust between them and negatively impact the self-esteem of the addicted nurse [16]. Nurses’ attitudes towards their substance-addicted colleagues may not only hinder the individual’s access to treatment but also detrimentally affect their social and professional life. An individual feeling stigmatized by coworkers may gravitate towards a circle of fellow substance users where they don’t feel ostracized. Nurses’ negative attitudes towards their colleagues they suspect are addicted can exacerbate the individual consequences of addiction [17, 18].

When nurses suspect drug addiction or a personality disorder, it should be clarified promptly to prevent stigmatization. If left untreated, the individual may exploit tolerance and goodwill, leading to a gradual decline in their work performance. Delayed detection of substance use can exacerbate problems [10]. It is the responsibility of nurses and hospital managers to identify employees with substance use early, refer them to treatment, ensure compliance with treatment, and develop institutional policies on this issue [3].

In the literature, numerous studies have been conducted examining nurses’ attitudes towards patients with substance use disorders [19–21]. Despite substance use rates among nurses being significant compared to the general population [22], there is a lack of sufficient research on colleagues’ attitudes towards narcotic drug addiction among nurses. This study is important in revealing colleagues’ perspectives on narcotic drug addiction among healthcare professionals. The study aimed to determine errors, omissions, and nurses’ attitudes and behaviors towards addicted colleagues in processes related to narcotic drug safety in hospitals. Additionally, the perceptions of nurses working with addicted colleagues were evaluated.

## Methods

### Procedure and samples

This study was conducted as a descriptive-cross-sectional model with nurses employed at a State Hospital located in a province in the northeastern part of Türkiye. The hospital provides services to adult patients, including outpatient services as well as internal medicine and surgical clinics, with intensive care units. It has a total of 460 beds, employing 550 nurses and 1300 staff members. Since 2005, the hospital has been managed according to patient and staff safety procedures, including narcotic drug safety, as part of the QHS standards adopted nationwide in Türkiye.

The population of the research consisted of 378 nurses working in the hospital between March 2023 and August 2023. However, due to the possibility that some of these nurses were on leave or refused to participate in the research, the sample calculation method was used. The sample of the research consisted of 191 nurses determined using the known sample calculation method (95% confidence interval, 5% margin of error). The sample of the qualitative part of the research; Among the nurses participating in the study, 39 nurses who had previous experience working with addicted healthcare workers were determined by the Analogous sampling method used in qualitative research designs. Out of the nurses who participated in the study, 39 responded to open-ended questions cotic drug safety, as part of the QHS adopted nationwide in Türkiye.

In this research, a mixed method consisting of quantitative and open-ended questions was used. The qualitative part of the study was based on descriptive *phenomenology theory*, with the aim of understanding in depth the experiences of nurses who had experience working with addicted healthcare professionals, among the participants who answered predetermined questions. Open-ended questioning technique was used within the scope of *unstructured interviews*, which is one of the qualitative research data collection techniques. This open-ended question, added to the end of the data collection form containing quantitative questions, was conducted immediately after the quantitative part.

With the data collection form containing quantitative data, the demographic information of nurses working in areas where narcotic drugs are used and their behaviors regarding narcotic drug safety management processes were determined. Nurses’ attitudes towards their addicted colleagues were evaluated with the Addictive Substance Attitude Scale. In the study, open-ended questions were added to the last section of the data collection form in order to determine the experiences of nurses working with addicted individuals and their opinions and feelings regarding these processes.

The data breakdown phase was carried out by writing down 39 answers obtained from open-ended questions. Common themes among similar expressions were identified. These common themes were identified as statements about trust, help and support offered to an addicted colleague, a normal working relationship, and acceptance of addiction. The expressions given according to these common concepts obtained are classified under 2 headings. The responses were grouped under the headings of trust-based attitudes towards addicted colleagues and behaviors towards addicted colleagues.

Prior to commencing the study, approval was obtained from the ethics committee of Ordu University Clinical Research Ethics Committee (2023 / Decision no. 68), and institutional permission was obtained from the Provincial Health Directorate. Participants were informed about the study in accordance with the Declaration of Helsinki, and their consent was obtained through the Informed Consent Form. Participation in the study was voluntary. Volunteers who wished to participate were required to complete a volunteer consent form, which outlined the purpose and methodology of the study as well as the rights of the volunteers regarding participation. The Personal Information Form and the Addictive Substance Attitude Scale were administered face-to-face to nurses by the researcher, and their data were collected. The completion of the forms took an average of 30–40 min.

The research sought answers to the following questions.

1. What are the problems experienced in carrying out narcotic drug safety processes in hospitals?
2. What is the attitude of nurses towards their addicted colleagues?
3. What are nurses’ opinions about their experiences with addicted colleagues?

### Data collection tools

Personal Information Form: It questioned nurses’ socio-demographic characteristics such as age, education, years of work, and number of children. Also, this form includes questions prepared to determine the management processes of narcotic drugs used in clinics, the problems experienced in these processes, and the attitudes and behaviors towards teammates who are addicted to these drugs.

Addictive Substance Attitude Scale (ASAS): The scale, whose validity and reliability analyzes were conducted by Kaylı et al. (2020) [23]. Measures attitudes towards people who use addictive substances, with a 5-point Likert scale (“I completely agree” = 1, “I somewhat agree” = 2, “I am undecided” = 3, “I disagree.” = 4, “I strongly disagree” = 5). An increase in the total score on the scale means having a more negative attitude towards individuals who use substances. Therefore, while calculating the total score, the scores of items other than items 7, 11, 12, 15, 19 and 20, that is, items with negative expressions, were reversed (this reversal process yields 1 = 5, 2 = 4, 3 = 3, 4 = 2, in the format 5 = 1).

Permission was obtained from the responsible researcher for the use of the scale. An increase in the total score on the scale means having a more negative attitude toward people who use addictive substances. The Cronbach Alpha coefficient of the scale was found to be 0.923. In the current study, the Cronbach’s Alpha value of the scale was found to be 0.872.

### Data analyses

The quantitative data analysis of the study was done in the Statistical Package for the Social Sciences (SPSS) 26.0 for Windows (SPSS, Chicago, Il, USA) package program. Whether the data was distributed normally or not was evaluated by the Skewness and Kurtosis coefficients being in the range of (-1) - (+ 1) [24]. Numbers, percentages and mean values and standard deviation (SD) were used for descriptive statistics. Independent Samples Test and the One- Way ANOVA test were used to compare the descriptive characteristics of the nurses and their scale scores. The relationship between some nurses’ variables and the total scale scores was examined with a multiple linear regression model. *P* < .001 and *p* < .05 were taken as levels of statistical significance.

Nurses who had previously worked with addicted individuals were asked an open-ended question about their attitudes and behaviors towards addicted colleagues. The data breakdown phase was carried out by writing down 39 answers obtained from open-ended questions. Common themes among similar expressions were identified. These common themes were identified as statements *about trust, help and support offered to an addicted colleague*, *a normal working relationship, and acceptance of addiction.* The expressions given according to these common concepts obtained are classified under two headings. The responses were grouped under the headings of *trust-based attitudes* towards addicted colleagues and *behaviors towards addicted colleagues.*

## Results

When examining the characteristics of the nurses participating in the study, it was observed that their average age was 36.58 ± 8.40, 89.0% were women, 85.3% had undergraduate or graduate education, 74.9% were married, and 72.3% had children. Additionally, 46.6% of the nurses worked in intensive care wards, 80.1% worked as clinical nurses, and 67.0% had ten or more years of work experience. Regarding drug usage, 69.6% of the nurses stated that they did not use drugs. Moreover, 85.3% reported that drug monitoring was conducted in the units they worked in, while 52.3% were unsure if there was an institutional policy regarding substance addiction. Furthermore, 55.5% mentioned receiving training on narcotic drug addiction, and 97.9% confirmed being on duty, with 96.3% stating that a post-seizure medication count was performed.

In terms of procedures related to missing drugs, 44.5% of the nurses notified the nurse in charge when detecting a missing drug in the count before the shift. Additionally, 69.1% sent half-used narcotic drugs to the pharmacy, and 72.8% routinely used the half-dose drug disposal form. Regarding awareness of procedures for healthcare workers addicted to narcotic drugs, 63.9% of the nurses stated they were not aware of such procedures. Furthermore, 61.3% indicated they would suggest their addicted friend to see a psychiatrist, and 79.6% had not worked with a drug addict before (Table 1).

**Table 1:** Distribution of Nurses’ Sociodemographic and Attitude Characteristics Towards Dependent Individuals

Variables	Results
	Mean ± SD
**Age**	36.58 ± 8.40
	**n(%)**
**Gender**	
Woman	170 (89.0)
Male	21 (11.0)
**Educational background**	
High School/Associate Degree	28 (14.7)
Undergraduate/Graduate	163 (85.3)
**Marital status**	
Married	143 (74.9)
Single	48 (25.1)
**Status of having children**	
Yes	138 (72.3)
No	48 (25.1)
**Unit of study**	
Indoor clinics/Palliative care	33 (17.3)
Surgical clinics	37 (19.4)
Intensive care	89 (46.6)
Emergency/other	32 (16.7)
**Position**	
Charge nurse	24 (12.6)
Clinic nurse	153 (80.1)
Other	14 (7.3)
**Year of study**	
0–9 years	63 (33.0)
10 years and above	128 (67.0)
**Substance use status**	
Yes (cigarette)	58 (30.4)
No	133 (69.6)
**Medicine follow-up status in the unit where you work**	
Yes	163 (85.3)
No	28 (14.7)
**Do you have an institutional policy regarding substance abuse?**	
Yes	62 (32.5)
No	29 (15.2)
He does not know	100 (52.3)
**Status of receiving narcotic drug addiction training**	
Yes	85 (44.5)
No	106 (55.5)
**Drug counting at seizure delivery**	
Yes	187 (97.9)
No	4 (2.1)
**Post-seizure medication count**	
Yes	184 (96.3)
No	7 (3.7)
**What do you do when you detect that there is missing medication in the count before the seizure?**	
I inform the nurse in charge	85 (44.5)
I’m keeping a record	34 (17.8)
I share the issue with my friends on duty and try to find out the reason.	72 (37.7)
**What do you do with the remaining half-used narcotic drugs?**	
I’ll save it for other patients	13 (6.8)
I will throw it in the bin	46 (24.1)
I will send it to the pharmacy	132 (69.1)
**Do you routinely use the half-dose medication disposal form?**	
Yes	139 (72.8)
No	52 (27.2)
**Do you know the procedure for a healthcare worker who is determined to be addicted to narcotic drugs?**	
Yes	69 (36.1)
No	122 (63.9)
**How are your social relations with your friend who is addicted to narcotic drugs?**	
I try not to meet socially.	36 (18.8)
In social life, I try to improve my communication and provide support.	24 (12.6)
I suggest that he go to a psychiatrist.	117 (61.3)
Other	14 (7.3)
**Have you ever worked with someone addicted to drugs?**	
Yes	39 (20.4)
No	152 (79.6)

Mean: Average, SD: Standard Deviation, n: Number, %: Percentage

The Addictive Substance Attitude Scale (ASAS) total score average of the nurses participating in the study was found to be 74.27 ± 14.70. Table 2 shows the comparison of some characteristics of nurses with their total scale scores. A significant difference was found between the total scores on the scale and the level of education of the nurses, the follow-up status of the drugs in the unit where they work, the status of receiving narcotic drug addiction training, and the routine use of the half-dose drug disposal form (*p* < .05). In the study, when the total score averages of the scale were compared with their educational status, it was determined that those who had a bachelor’s degree or higher had a higher scale score than those who graduated from high school. It was determined that the scale scores of nurses who reported that medication monitoring was not done in the unit in which they worked were higher than those who reported that medication monitoring was done. Additionally, the average score of nurses who received narcotic drug addiction training was found to be higher than those who did not receive training (Table 2).

**Table 2:** Comparison of Some Characteristics of Nurses with Scale Total Scores

Variables	*n* (%)	Total Score Average (Mean ± SD)	Test value *P* value**
**ASAS total score average**: 74.27 ± 14.70 **Age average**: 36.58 ± 8.40			
**Age group**			
18–25	17(8,3)	72.11 ± 13.28	F = 1.191 *p* = .203
26–35	77(40,3)	73.15 ± 13.51	
36 and above	97(50,8)	75.54 ± 15.82	
**Gender**			
Woman	170(89.0)	73.78 ± 14.44	t=-1.327 *p* = .186
Male	21(11.0)	78.28 ± 16.46	
**Educational background**			
High School/Associate Degree	28(14.7)	65.96 ± 14.63	**t=-3.323** *p* = .001*
Undergraduate/Graduate	163(85.3)	75.70 ± 14.28	
**Marital status**			
Married	143(74.9)	74.00 ± 14.30	t=-0.438 *p* = .662
Single	48(25.1)	75.08 ± 15.97	
**Status of having children**			
Yes	138(72.3)	73.53 ± 14.76	t=-1.125 *p* = .262
No	53(27.7)	76.20 ± 14.50	
**Substance use status**			
Yes (cigarette)	58(30.4)	74.56 ± 15.56	t = 0.180 *p* = .857
No	133(69.6)	74.15 ± 14.37	
**Medicine follow-up status in the unit where you work**			
Yes	163(85.3)	73.27 ± 13.80	**t=-2.296** *p* = .023*
No	28(14.7)	80.10 ± 18.36	
**Status of receiving narcotic drug addiction training**			
Yes	85(44.5)	76.75 ± 13.81	**t = 2.102** *p* = .037*
No	106(55.5)	72.29 ± 15.15	
**Working with drug addicts**			
Yes	39(20.4)	75.53 ± 17.27	t = 0.599 *p* = .550
No	152(79.6)	73.95 ± 14.01	
**Knowing the dependent employee follow-up procedure**			
Yes	69(36.1)	73.33 ± 15.50	t=-0.666 *p* = .506
No	122(63.9)	74.81 ± 14.26	
**Routine use of the half-dose medication disposal form**			
Yes	139 (72.8)	72.56 ± 13.79	**t=-2.680** *p* = .008*
NO	52 (27.2)	78.86 ± 16.15	
**Unit of study**			
Internal clinics/Palliative care	33(17.3)	72.12 ± 14.50	F = 0.369 *p* = .775
surgical clinics	37(19.4)	75.75 ± 14.10	
Intensive care	89(46.6)	74.53 ± 14.35	
Emergency/Other	32(16.7)	74.06 ± 16.85	
**How to treat a co-worker with narcotic drug addiction**			
Keep A Record	107(56.0)	73.90 ± 15.24	F = 0.138 *p* = .871
Don’t Talk To Him	32(16.8)	74.03 ± 15.07	
Other	52(27.2)	75.19 ± 13.53	

Mean: Average, SD: Standard Deviation, t: t test in independent groups, F: ANOVA test, *p* < .05

In line with the literature, the relationship between some nurses’ variables and total scale scores was examined with a multiple linear regression model (Table 3). In the analysis of some nurses variables, it was seen that there was a significant model in the evaluation of model goodness of fit (F/p) regression coefficients (R/R^2^) (*p* < .01). 11.3% of the variance in the dependent variable of the Addictive Substance Attitude Scale was explained by the independent variables (R^2^ adjusted = 0.113). It was determined that the educational status of the nurses and their routine use of the half-dose drug disposal form were statistically significant predictors in a positive direction, and the status of nurses receiving narcotic drug addiction training was a statistically significant predictor in the negative direction (*p* < .01, Table 3).

**Table 3:** Multiple Linear Regression Analysis Model Of Scale According to some variables

Scale	Variables						95,0 Cl		Model fit
		B	SE	β	t	*p*	Lower	Upper	Adj. R2 = 0.113 F = 9.028
Scale total score	(Constant)	55.405	6.686	-	8.287	0.000	42.215	68.594	
	Educational background	10.205	2.754	0.255	3.706	**0.000**	4.772	15.637	
	Status of receiving narcotic drug addiction training	-5.471	1.961	− 0.192	-2.790	**0.006**	-9.339	-1,603	
	Routine use of the half-dose medication disposal form	6.453	2.207	0.201	2.923	**0.004**	2.098	10.808	

Adj.R2: Adjusted R square; B: Partialregressioncoefficient; β: Standard partial regression coefficient; 95% CI: 95% confidence interval

In the study, 39 nurses responded affirmatively to the semi-structured question “Have you ever worked with a healthcare professional who you know is addicted?” When asked to summarize their approaches and experiences in a few sentences, the following responses were obtained:

Nurses’ attitudes towards addicted colleagues:

Nurses reported that when working with a healthcare professional addicted to drugs, they initially attempted to assist their addicted colleagues individually. Subsequently, they distanced themselves from the environment and exercised extra caution. They mentioned that they secured the narcotic medicine cabinet in the presence of the addicted colleague at the workplace to prevent access to drugs.

Nurses’ attitudes and behaviors towards addicted colleagues:

They indicated that they endeavored to support their colleagues known to be addicted by encouraging them to seek treatment, recommending professional help, maintaining communication, providing ongoing support throughout the process, documenting incidents to inform management, and continuing their friendships as long as it did not compromise their own well-being.

## Discussion

In clinics, the management of narcotic drugs is carried out according to a prescribed procedure determined by QHS standards. This procedure encompasses the prescription of the drug, its request from the pharmacy, stages of transportation, labeling, storage, administration, effects on the patient, and disposal of excess doses. These processes are carried out primarily by nurses. Continuous in-service training and on-the-job training must be repeated to ensure smooth progression of the process. To identify situations where drug safety is compromised, safety reporting systems have been established. However, due to the neglect that comes from the constant repetition of the same tasks or a busy work pace, some steps in this process may occasionally be overlooked.

In the hospital where the research was conducted, drug management has been carried out under quality standards since 2005. After the narcotic drugs are prescribed by the physician, they are personally received by the nurse on behalf of the patient and kept in a locked cabinet. Drugs are counted at every shift change, and the drugs used are recorded under the patient’s name. The remaining doses of drugs requested in half doses are destroyed with the assistance of a pharmacist using a half-dose drug disposal form.”

The majority of participating nurses (85.3%) indicated that drug tracking is performed in their units. Almost all of them (97.7%) reported counting and delivering narcotic drugs before and after their shifts. From this perspective, it can be said that nurses adhere to protocols in the management of narcotic drugs within the framework of healthcare quality standards. However, the disposal of remaining doses after drug administration is also an important part of this process. In the study, 6. 8% of nurses mentioned storing the remaining dose for use on another patient or the same patient. While storing the remaining doses with good intentions may seem logical, it poses a risk of misuse for individuals with addiction. Especially, these remaining doses left unnoticed during shift changes can be used for unintended purposes. To control the disposal of remaining doses, a half-dose disposal form has been developed within the framework of quality standards. In the study, 27. 2% of nurses stated that they did not fill out the half-dose disposal form. This form is used to control the remaining doses of narcotic drugs given to patients. In the study, 24. 1% of nurses mentioned throwing away the remaining drugs.

In Dadak et al.‘s study [25], it was observed that anesthesia specialists (87%) and psychiatry workers (72%), who work in areas where narcotic drugs are more frequently used, had the highest rates of addiction among healthcare personnel. A study conducted on the regulation of narcotic drugs in a university hospital revealed that narcotic drugs are prepared before procedures, especially in operating theater units, and excess products are obtained from the pharmacy [26]. While these are well-intentioned initiatives aimed at expediting medical procedures by stockpiling drugs before their definitive use, they may inadvertently facilitate access to and misuse of drugs by individuals struggling with addiction [25].

According to QSH standards, unused doses of narcotics should be disposed of with a written report under the supervision of the responsible personnel responsible for narcotic drug monitoring. When looking at the literature, there are not many studies related to the safety of narcotic drugs. However in a study conducted in Canada, 70 reports related to narcotic drug safety were observed in a 442-bed healthcare institution [27]. All employees in the participating hospital in our study reported counting medications during shift turnovers. Of the nurses participating in the study, 44. 5% reported informing the responsible nurse when they detected missing drugs, 17. 8% documented the incident and 37. 7% investigated and attempted to find the missing drug. However during the period of the study, no drug safety reports were found in the institution. In terms of drug safety, the activation of the safety reporting system, conducting root cause analysis and initiating corrective actions through the creation of official statistics are important. The disposal of unused medications may not have been documented on the safety reporting form, as it may have been perceived not to pose a threat to patient safety. In their examination of approximately two years of retrospective safety reporting records at a public hospital, İncesu and Orhan (2018) found no data related to medication safety [28]. Written reporting during the provision of healthcare services contributes to the establishment of a reporting culture within the institution, enabling the identification of the root cause of errors and guiding improvements in the necessary direction [29]. Therefore, it is crucial to design patient and employee safety reporting systems in a way that is understandable to all employees, adapt them to the system, and provide training to employees on reporting systems [28].

The disposal of unused medications may have been overlooked, assuming it did not pose a threat to patient safety, thus resulting in the security reporting form not being filled out. However, considering the potential risk for employees and other individuals with substance dependence, the disposal of unused medications should be assessed as a preventive measure. When İncesu and Orhan (2018) examined approximately two years of retrospective security reporting records in a public hospital, they found no data regarding medication safety [28].

In the country where the study was conducted, there is no official data on narcotic drug use among healthcare professionals. According to a presentation by the Emergency Medicine Specialists Association (ATUDER) on “Substance Use and Suicide Risk in Emergency Service Employees,” 50 healthcare professionals were found dead in their rooms due to drug overdose over a 10-year period [30]. Moreover, a media search conducted by the BBC between October and June 2022 found that at least 6 healthcare professionals in the anesthesia, emergency services, or intensive care branches suspiciously lost their lives [31, 32]. These professionals may have obtained drugs from the hospital, wards, or leftover doses given to patients.

Addiction to narcotic drugs is also a workplace safety issue. The treatment processes of nurses identified as addicted to opioid or anesthetic substances include acceptance and initiation of treatment, providing social and psychological support to the individual, and rehabilitation. The attitudes and behaviors of nursing colleagues are crucial at all stages. Early recognition of addicted individuals, providing support during treatment, and effectively managing the process during rehabilitation are important for reintegrating the addicted individual into society. In this study, while a percentage of nurses received training on the safety of narcotic drugs, 55. 5% did not receive education on substance addiction. The research revealed a significant difference in ASAS scores between nurses who received training on drug addiction and those who did not. Trained nurses exhibited more negative attitudes. This situation may be attributed to the fact that the content of the training only focused on narcotic drug safety.

A study conducted until 2020, which analyzed the meta-analysis of medication safety training conducted under pharmacist supervision, revealed that the training provided covered the stages of procurement, preparation, and administration of medications, but did not specifically include training on narcotic drug management [33]. Additionally, within the scope of these trainings, healthcare professionals should be provided with awareness on the misuse and addiction of narcotic drugs [34].

Supporting individuals with addiction socially fosters their sense of belonging to a community and helps them believe that they are valued, protected, accepted, and respected in an environment where they feel loved. In a study conducted with cocaine-dependent individuals, it was found that perceived social support positively impacted psychological well-being and reduced anxiety levels [35]. During the addiction process, individuals who receive help from friends or colleagues do not struggle with accepting their identity and self-concept. Social support enables individuals to cope more effectively with feelings of helplessness and seek new solutions [36]. Therefore, the attitude of colleagues towards a nurse suspected of addiction plays a significant role in their acceptance of treatment and recovery.

In the study, the ASAS scale was used to measure nurses’ attitudes towards addicted colleagues. The total ASAS score average in the research was determined as 74.27 ± 14.70. Comparing this result with a study by Kayli et al. with individuals in the community (*n* = 222), where the average ASAS score was 92.15 [23], the average ASAS score of nurses in our study is lower. An increase in the total score on the scale indicates a more negative attitude towards individuals who use substances. It can be said that the attitudes of the nurses in the study are more positive compared to the results obtained in the study by Kayli et al. [23]. Another study investigating the attitudes of emergency nurses towards addicted individuals found that they exhibited negative attitudes towards maintaining social distance from addicted individuals. The attitude scale scores identified in the study by Pilge and B. Arabacı (2016) (Mean: 49.43 ± 19.59) indicate that emergency nurses have a more negative attitude compared to the results of the current study [37].

This difference may be due to demographic variations. It can be said that nurses are less biased toward addicted individuals compared to the general population [21]. Some research in the literature supports the results of our study by showing that the attitudes of healthcare professionals toward addicted individuals are more positive [38–40]. Broadu and Evans identified factors such as gender, age, education, religious beliefs, and history of addiction treatment as influencing attitudes toward addicted individuals [41]. In this study, gender, age, years of experience, and history of addiction did not affect the ASAS score.

In the literature, it has been observed that age and gender do not affect attitudes both in society and among healthcare professionals similar to the results of the study [42–44]. Only individuals with a bachelor’s degree or higher exhibited a higher ASAS score. It was noted that individuals with higher levels of education demonstrated elevated ASAS scores. It is hypothesized that exposure to education regarding drug addiction during their academic pursuits may amplify biases. Incorporating addiction-related subjects into school curricula or educational settings often relies on oversimplified and historical perspectives. A comprehensive health education should encompass the significance of social determinants of health, recognizing that addiction entails complex biopsychosocial processes that cannot be adequately addressed in isolation [45, 46]. Consequently, educational interventions solely focusing on depicting addiction’s consequences and passing judgment may exacerbate bias against individuals struggling with addiction. The investigation revealed no significant disparities in scale scores between those who had prior experience working with individuals with addiction and those who had not.

In the study nurses, when asked open-ended questions about their experiences working with individuals struggling with addiction, expressed that they continued their work as if “such a situation did not exist.” This sentiment is supported by Bettinardi & Bologeorges’ (2011) study, where 57% of nurses stated that they would not report suspicions of substance use among their colleagues [47]. Dependent healthcare workers are still not adequately assessed and continue to receive insufficient treatment for addiction and substance dependency [4].

Managers who do not establish procedures for detecting and monitoring narcotic drug addiction in their institutions, along with employees who fail to implement these procedures, may overlook the presence of an addicted employee. Fear of damaging the institution’s reputation, causing harm to the employee, termination of employment, or protecting colleagues may prevent reporting regarding the addicted individual. During this process, the addiction of an individual who fails to recognize the need for help may worsen. Early detection and initiation of treatment are crucial as addiction tends to become more chronic over time. Even if the job performance of the addicted nurse has not yet deteriorated, directing them towards treatment, with a focus on alcohol and substance addiction, is imperative [10]. Acceptance and engagement in treatment represent significant steps in combating addiction. The attitudes of those around addicted individuals influence both the acceptance phase and the rehabilitation process [48]. Negative societal attitudes towards addicted individuals can lead to their isolation [49]. In the study, 18.8% of nurses stated they would not socialize with addicted individuals, while 12.6% expressed willingness to improve communication and offer support for their treatment. Early detection and referral to treatment for a nurse suspected of addiction are critical for fostering self-confidence, overcoming denial, and encouraging initiation and continuation of treatment [49, 50]. American Nurses Association (ANA) is calling on professional nurses to support their addicted colleagues, ensure access to appropriate treatment, and advocate for fair treatment in institutional practices [51].

In response to open-ended questions, some nurses in the research mentioned experiencing trust issues with the addicted individuals they worked with. Approaching addicted individuals with bias, behaving as if drug theft could occur at any moment, not only impacts the self-confidence of addicted individuals but also contributes to their social exclusion. Individuals who feel alienated from society and isolated may seek solace among other addicted individuals who have encountered similar discrimination, thereby reinforcing each other’s behaviors and potentially normalizing addiction. Consequently, the individual may be less inclined to seek help.

In another study examining the perspectives of healthcare professionals on substance addiction, it was revealed that they preferred not to be in the same social environments as patients using substances. A systematic review by Van Boekel et al. highlighted that negative attitudes among healthcare professionals toward patients with substance use disorders were widespread and had implications for treatment outcomes. Interestingly, in the study mentioned, there were no significant differences in scale scores between those who had prior experience working with an addict and those who had not [14].

## Theoretical implications

One significant aspect that sets nurses apart from other hospital staff is their easier access to narcotic drugs. Obtaining a narcotic drug, creating addiction with this drug, or sustaining this addiction can be easier. Nurses’ attitudes and behaviors towards their colleagues who are addicted to narcotic drugs demonstrate their efforts to support addicted individuals and their willingness to direct them towards treatment. These attitudes are important for the early detection of addiction and for supporting addicted individuals during the rehabilitation process. Nurses play a crucial role in combating addiction by encouraging their addicted colleagues to seek treatment, recommending professional help, maintaining communication, and providing support at every stage of the process.

### Managerial implications

In the tracking of narcotic drug management processes, drug safety reporting systems are crucial. During the study period, it was found that the institution where the research was conducted did not have drug safety reports, including those related to narcotic drug management processes. For drug safety, it is important to activate the safety reporting system, conduct root cause analyses, and take corrective actions based on official statistics. Considering the potential risks to employees and other individuals with substance dependence, the disposal of unused drugs or identifying missing drugs can be evaluated as preventive measures. Therefore, careful execution of narcotic drug tracking processes is vital for the early detection of addicted individuals, prevention of overdose deaths among healthcare workers, and ensuring safe patient care. Comprehensive training related to narcotic drug management should include legal regulations, safe storage and distribution measures, proper dosage and administration, as well as the use and intervention of narcotic drugs in emergencies. Additionally, these trainings should cover the causes of drug addiction, symptoms observed in addicted individuals, approaches to dealing with addicted individuals, and even case studies.

### Limitations

This study had some limitations. First, this study used self-report measurement instruments, which can introduce some form of response bias. Secondly, since this study was conducted in a province located in the northeastern part of Türkiye the results cannot be generalized. Third, since the study was cross-sectional, causality could not be determined. Therefore, caution is recommended when interpreting the study results. Despite these limitations, the study had its strengths. This study is valuable in terms of evaluating the attitudes and behaviors of nurses, a very special group with a large majority in the healthcare system, toward individuals with narcotic drug and substance addiction in many aspects and raising awareness among nurses about this issue. Future research could enhance the generalizability of the findings by including larger sample groups and participants from diverse geographical regions and cultures to assess nurses’ perspectives. Additionally, future studies should aim to improve the accuracy of results by utilizing objective measurement methods alongside subjective measurement tools. However, considering the limitations of the cross-sectional design, future research is recommended to prefer longitudinal or experimental designs to better understand causal relationships. Consequently, the limitations of this study should be taken into account for future research, employing more comprehensive methods and increasing the generalizability of results.

## Conclusions

This study addresses the attitudes of colleagues towards addicted nurses, which is a significant aspect of the narcotic drug management processes in healthcare institutions, aimed at ensuring the safe and effective management of narcotic drugs. The findings indicate that protocols established for the correct and safe use of narcotic drugs are generally followed. However, deficiencies in the disposal of remaining doses of drugs after administration may potentially increase the risk of misuse. Additionally, it has been emphasized that addiction related to the use of narcotic drugs among healthcare workers and its consequences constitute a serious issue. In this context, the education and awareness-raising of healthcare workers are of critical importance in ensuring the safety of narcotic drugs and preventing addiction. The findings also reveal that some nurses experience distrust when working with addicted colleagues, while others continue their work as if such a situation does not exist. These attitudes may jeopardize patient safety, lead to the neglect of health issues among addicted individuals, and hinder their access to effective treatment. Therefore, increasing awareness of addiction among healthcare workers and adopting a sensitive attitude towards this issue are important. Furthermore, as highlighted by the study, existing policies and practices in this regard need to be strengthened. This can enhance the effective management of narcotic drugs while improving patient and staff safety and support.

## Data Availability

He datasets used and/or analysed during the current study are available from the corresponding author on easonable request.

## Declarations

### Declarations

The authors declare that this article has not been presented in any journal or conference before.

## Abbreviations

USA: United States of America
ASAS: Addictive Substance Attitude Scale
ATUDER: Association of Emergency Medicine Specialists
BBC: British Broadcasting Corporation
HCS: Health Quality Standards
ANA: American Nurses Association
